# Resveratrol inhibits the inflammatory response and oxidative stress induced by uterine ischemia reperfusion injury by activating PI3K-AKT pathway

**DOI:** 10.1371/journal.pone.0266961

**Published:** 2022-06-24

**Authors:** Ying Wang, Yong Wu, Shu Peng

**Affiliations:** 1 Reproductive Medicine Center, Jingzhou Hospital Affiliated to Yangtze University, Jingzhou, Hubei, China; 2 Reproductive Medicine, Maternal and Child Health Hospital of Hubei Province, Wuhan, Hubei, China; Zagazig University, EGYPT

## Abstract

Uterus transplantation is a complex surgical procedure. Uterine ischemia reperfusion (I/R) injury that occurs during this process may cause a loss of function of the uterus and the failure of transplantation. Resveratrol (RSV) is a naturally occurring polyphenol found abundantly in the skin of grapes and red wine, and it has also been used as a dietary supplement in clinical practice. RSV possesses strong anti-inflammatory and anti-oxidative effects, and exhibits a role in immune system regulation. However, the role of RSV in protecting the uterus against I/R-induced injury is yet to be fully elucidated. The aim of the present study was to investigate the effects and mechanisms underlying RSV in I/R-induced uterus injury. A total of 48 Sprague-Dawley rats were randomly divided into four groups: Control (sham operation) group, the uterus I/R group, the 20 mg/kg RSV-pre-treated I/R (I/R+RSV/20) group and the 40 mg/kg RSV-pre-treated I/R (I/R+ RSV/40) group. A regular I/R model was established to induce uterus injury in rats. RSV at 20 or 40 mg/kg was intraperitoneally administrated into rats in both I/R+ RSV groups once per day for five consecutive days prior to ischemia. The control and I/R only groups received an intraperitoneal injection of the vehicle (ethanol) for the same period prior to ischemia. Samples from blood and uterine horns were collected 3 h after reperfusion. Changes in the levels of malondialdehyde, interleukin (IL)-6, tumor necrosis factor-α, IL-10 and IL-37 were determined using ELISA, the activity levels of myeloperoxidase, catalase, and superoxidase dismutase were also determined using ELISA, the protein expression levels of PI3K, phosphorylated (p)-PI3K, AKT and p-AKT were determined using western blot analysis, and the uterine histology was investigated using H&E staining. Results of the present study demonstrated that treatment with RSV increased the capacity of antioxidants and suppressed uterine oxidative injury induced by I/R. Moreover, treatment with RSV decreased the levels of pro-inflammatory cytokines and increased the levels of anti-inflammatory cytokines. In addition, RSV promoted the phosphorylation of PI3K and AKT, thus activating the PI3K-AKT signaling pathway. Therefore, administration of RSV prior to uterine I/R effectively alleviated inflammatory response and oxidative stress via activation of the PI3K-AKT pathway, suggesting that RSV may play a protective role in I/R-induced uterus injury.

## 1. Introduction

Uterus transplantation (UTx) is the only treatment available for patients with uterine infertility to conceive, which affects 3–5% of the total female population worldwide [1]. Uterine infertility is often caused by congenital uterine hypoplasia (MRKH syndrome), hysterectomy or severe intrauterine adhesions [1], meaning that these patients can only have children through surrogacy or adoption. However, surrogacy is illegal in numerous countries, and adoption may be considered a potential factor causing child trafficking. To date, a total of 52 human UTx cases have been carried out worldwide [2], with only 14 cases being successfully delivered via the transplanted uterus, demonstrating a very low rate of success.

Ischemia reperfusion (I/R)-induced tissue damage causes morbidity and mortality in many diseases, including acute coronary syndrome, acute chest syndrome, acute lung injury, sleep apnea and ischemic stroke [3]. I/R injury is also a major challenge during solid organ transplantation, leading to acute graft failure and early graft rejection [3]. The loss of balance in metabolic supply and demand in ischemic organs leads to severe hypoxia and microvascular dysfunction, while subsequent reperfusion further activates the innate and adaptive immunoreaction, as well as cell death [4]. Treatment approaches for I/R have been developed to increase ischemia tolerance or to alleviate reperfusion injury, including the use of prolyl hydroxylase inhibitors [5] or adenosine receptor agonists [6]. During UTx surgery, uterine I/R injury causes disordered energy metabolism, the excessive production of free radicals and the infiltration of inflammatory cells, suggesting that I/R may act as one of the critical factors affecting UTx success and functional reconstruction.

Resveratrol (RSV) is a naturally occurring polyphenol found in berries, peanut peels and grapes. It is beneficial for human health due to its anti-inflammatory, anti-oxidative, anti-aging and anti-tumor effects [7]. Results of previous clinical trials have demonstrated that RSV supplementation is safe and well tolerated at a dose of up to 5 g [8]. The bioavailability and pharmacokinetics of RSV depend on many factors, including the dose ingested, the particle size and the role of gut microbiota in RSV metabolism [9]. Recent studies have found that RSV may resist oxidative damage, therefore demonstrating potential as a reactive oxygen species (ROS) scavenger [10]. RSV is also an effective antioxidant that can regulate the activity of antioxidant enzymes via transcriptional regulation [11]. RSV significantly reduces I/R-induced organ damage, including kidney, liver, heart and small intestine [10]. However, the protective role and mechanism underlying RSV against I/R-induced uterus injury is yet to be fully elucidated. In the present study, a rat uterine I/R injury model was established [12], the serum and tissue levels of inflammatory cytokines and oxidative stress-related molecules were measured, the activation of PI3K-AKT signaling was evaluated, and the protective effect and mechanisms underlying RSV were discussed. The present study may provide a novel theoretical basis for the prevention and treatment of I/R-induced uterine injury. However, studies using large animals or randomized controlled trials are also required to expand on current knowledge and evaluate the rationale for clinical use.

## 2. Materials and methods

### 2.1. Animals

Adult female Sprague-Dawley rats (age, 8 weeks) weighing between 200–250 g were commercially purchased. Rats were housed under standard conditions of temperature (23 ± 2°C), relative humidity (65 ± 5%) and a 12-h light/dark cycle. Food and water were provided *ad libitum*. Animals received humane care according to the Guide for the Care and Use of Laboratory Animals of National Institutes of Health. The animal care and experimental procedures were approved by the Animal Policy and Welfare Committee (approval no. 20190914–122) of Jingzhou Hospital affiliated to Yangtze University. At the end of the experimental period, rats were euthanized by carbon dioxide asphyxiation at a flow rate of 30% chamber vol/min.

### 2.2. RSV treatment

A total of 48 rats were randomly and evenly divided into four groups: i) The control (sham operation) group; ii) the uterine I/R group; iii) the 20 mg/kg RSV-pre-treated I/R (I/R + RSV/20) group; iv) the 40 mg/kg RSV-pre-treated I/R (I/R + RSV/40) group. RSV (Sigma-Aldrich; Merck KGaA) was dissolved in 25% ethanol and subsequently diluted with sterile saline. The prepared RSV at 20 or 40 mg/kg was intraperitoneally injected into rats once per day for five consecutive days prior to ischemia. Rats in the control group and I/R only group received an intraperitoneal injection with ethanol for the same period prior to ischemia. A rat uterine I/R model was established as follows: Rats were anesthetized with 60 mg/kg ketamine hydrochloride and 10 mg/kg xylazine intramuscularly. The surgical areas were subsequently shaved and disinfected with povidone-iodine solution, followed by clamping of the lower uterine horn and uterine artery using smooth vascular clips [12]. After 1 h uterine ischemia, clamps were removed and reperfusion was allowed for 3 h. The abdominal incision was closed with a 3–0 silk suture during reperfusion [12]. After the total 4 h I/R period, rats were euthanized by carbon dioxide asphyxiation at a flow rate of 30% chamber vol/min. The serum and both uterine horns of rats were harvested for subsequent experiments. All surgical procedures were performed under sterile conditions.

### 2.3. Biochemical analysis

The concentration of malondialdehyde (MDA) in the serum and uterine tissues was measured using thiobarbituric acid chromatometry [13] at a wavelength of 450 nm using a MDA Assay kit (Beyotime Institute of Biotechnology).

Catalase (CAT) activity was determined spectrophotometrically using ammonium molybdate to produce a yellow complex with H_2_O_2_ [14]. Kinetic analysis was performed at 405 nm on an Ultrospec 2000 spectrophotometer. CAT activity was defined as mmol H_2_O_2_ reduced per min (mmol H_2_O_2_/min).

The activity of superoxidase dismutase (SOD) in the serum and uterine tissues was measured using the xanthine oxidase method [15], with kits supplied by Beyotime Institute of Biotechnology. Wavelengths were read at 560 nm on a microplate reader. Myeloperoxidase (MPO) activity was measured using a spectrophotometric method [16] with 3, 3–5,5 tetramethyl benzine as the substrate, and the light absorbance was read at 655 nm over a period of 5 min.

The values of CAT, SOD and MPO activity were presented as U/μl or l of serum or U/μg or g of tissue. All detection procedures were strictly in accordance with the kit instructions.

### 2.4. ELISA assay

ELISA kits for interleukin (IL)-6, IL-10, IL-37 and tumor necrosis factor (TNF)-α (R&D Systems Inc.) were used to determine the concentration of each cytokine both in the plasma and uterine tissues. A 100-ul aliquot of the supernatant from each well was taken for measurement. The concentration of each cytokine in the supernatant was standardized to the cell protein concentration in the respective well. The absorbance was measured at 450 nm using a Model 550 Microplate Reader (Bio-Rad Laboratories, Inc.). Experiments were repeated four times using different batches of rats.

### 2.5. Western blot analysis

Frozen uterine tissues were homogenized in RIPA buffer [2 mM EDTA, 0.1% (w/v) SDS, 50 mM Tris-HCl, 150 mM sodium chloride, 1% Triton X-100 and 1% (w/v) sodium deoxycholate], and freshly supplemented with protease and phosphatase inhibitor cocktails (Sigma-Aldrich; Merck KGaA) on ice. Lysates were harvested following centrifugation at 12,000 x g for 30 min at 4°C. Protein concentration was determined using a Bradford assay. Total proteins were boiled for 5 min at 100°C. A total of 30 μg protein for each sample were loaded onto a 10% SDS-polyacrylamide gel and then subjected to electrophoresis at 100 V for 120 min. Separated proteins were subsequently transferred onto PVDF membranes (Millipore Sigma) for 90 min. Following blocking for 1 h in Tris-buffered saline with Tween-20 (TBS-T) with 5% non-fat milk, the PVDF membranes were washed four times in TBS-T (15 min each time) and incubated with primary antibodies (Table 1) overnight at 4°C. Following primary incubation, membranes were washed again four times in TBS-T and incubated with horseradish peroxidase (HRP)-conjugated secondary anti-rabbit or anti-mouse antibodies (1:1000; Santa Cruz Biotechnology, Inc.) for 1 h at room temperature. The membranes were subsequently washed again four times with TBS-T and visualized using enhanced chemiluminescence (ECL kit; Millipore Sigma). Protein bands were scanned and analyzed using Quantity One Image Analysis Software (Bio-Rad Laboratories, Inc.). Mouse anti-β-actin antibody was used to detect β-actin as an internal loading control. Western blot analysis was carried out to determine protein expression levels of PI3K, phosphorylated (p)-PI3K, AKT and p-AKT in the RSV/40 group, but not in the RSV/20 group.

**Table 1:** Primary antibodies.

Target gene	Catalog no.	Host	Vendor	Dilution
**PI3K**	ab191606	Rabbit	Abcam	1:1000
***p*-PI3K**	ab182651	Rabbit	Abcam	1:500
**AKT**	2920	Mouse	Cell signaling technology	1:2000
***p*-AKT**	9271	Rabbit	Cell signaling technology	1:1000
**β-actin**	sc-8432	Mouse	Santa cruz biotechnology	1:200

### 2.6. H&E staining

The uterus samples were removed and immediately placed in 10% formalin solution, then embedded in paraffin blocks. Samples were sectioned into 5 μm-thick slices, stained with H&E for histopathological examination, and visualized under a light microscope (Olympus Corporation).

### 2.7. Statistical analysis

Statistical analysis was conducted using Graph Pad Prism 6.0.1 (GraphPad Software Inc.). The data are expressed as the mean ± standard deviation. One-way ANOVA followed by Turkey’s post hoc test was applied for multiple comparisons. P<0.05 was considered to indicate a statistically significant difference.

## 3. Results

### 3.1. RSV pre-administration attenuates uterine IR-induced oxidative stress in the serum and uterus

**Fig 1:**
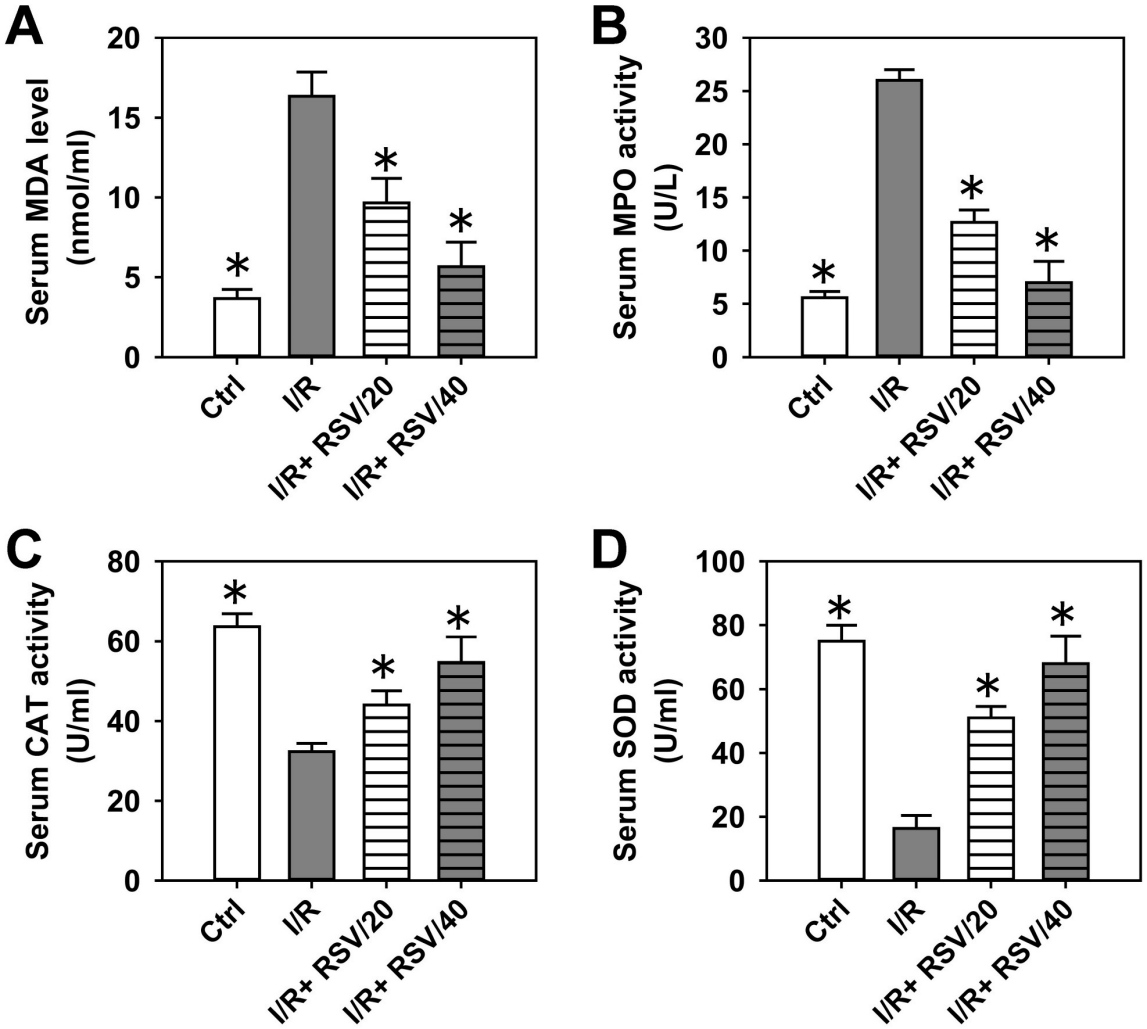
The effect of RSV on the oxidative stress induced by uterine I/R in rat serum. (***A***) The content of MDA in rat serum in control, I/R, I/R+RSV/20, and I/R+RSV/40 groups. (***B-D***) The activities of MPO (***B***), CAT (***C***) and SOD (***D***) in rat serum in each group. Bars represent means ± SD, n = 3. *P< 0.05 *vs*. I/R group.

In order to evaluate the protective effect of RSV against oxidative stress, several parameters in the serum were detected, including the concentration of MDA, and the activity levels of MPO, CAT and SOD (Fig 1). As demonstrated in Fig 1A, I/R significantly induced the serum concentration of MDA by 4-fold, while pre-administration of RSV notably decreased the I/R-induced MDA level by 40–70%. Moreover, I/R markedly enhanced the serum MPO activity by ~4.5-fold, while RSV pre-treatment inhibited I/R-induced MPO activation by 50–70% (Fig 1B). By contrast, I/R markedly inhibited both CAT and SOD activity in the serum by 50 and 75%, respectively, while RSV significantly promoted the restoration of CAT and SOD activity by1.4–1.6-fold and 3-4-fold in a dose-dependent manner (Fig 1C and 1D).

**Fig 2:**
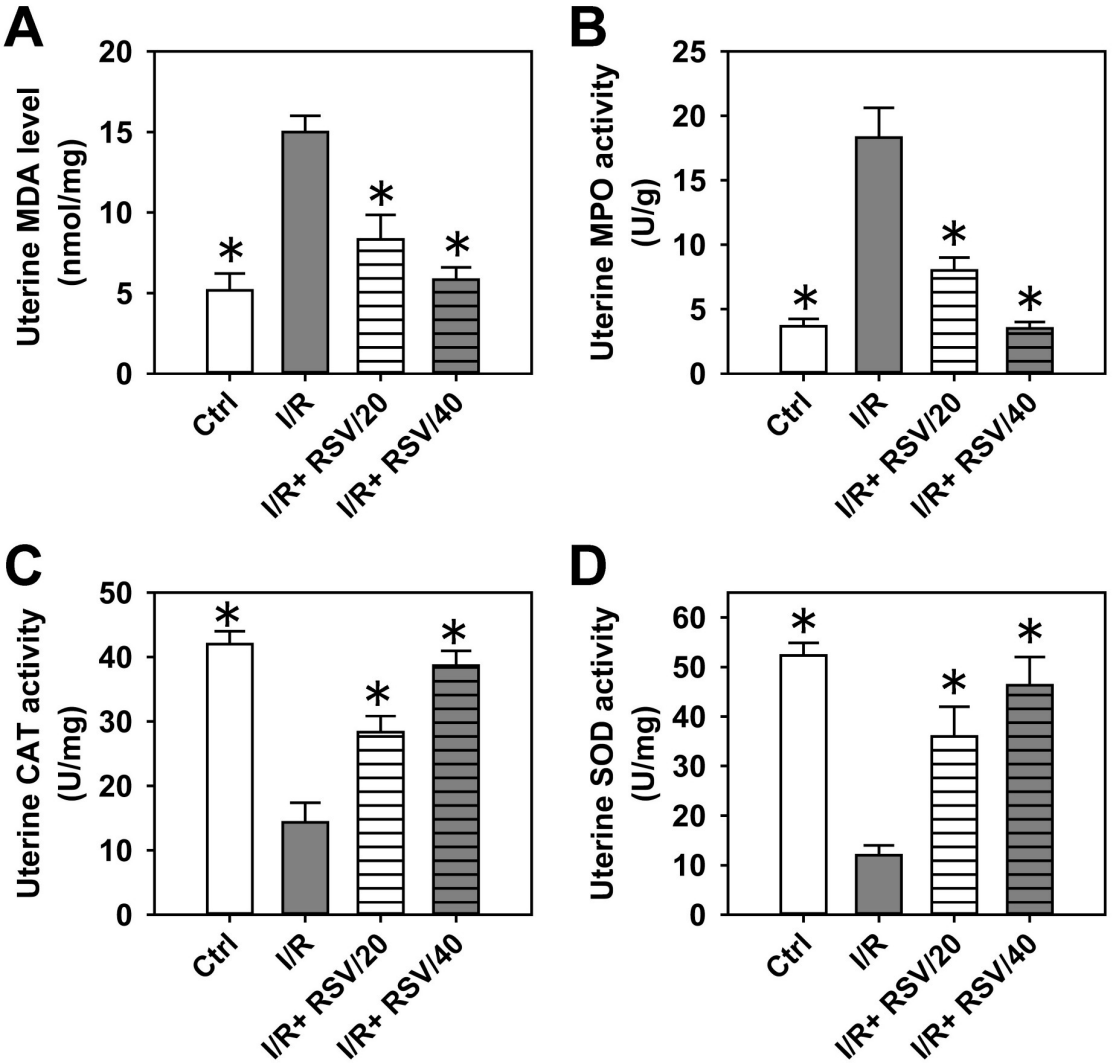
The effect of RSV on the oxidative stress induced by uterine I/R in rat uterus. (***A***) The content of MDA in rat uterus in control, I/R, I/R+RSV/20, and I/R+RSV/40 groups. (***B-D***) The activities of MPO (***B***), CAT (***C***) and SOD (***D***) in rat uterus in each group. Bars represent means ± SD, n = 3. *P< 0.05 *vs*. I/R group.

Subsequently, the protective effects of RSV against oxidative stress were determined in tissues. As shown in Fig 2A, RSV pre-treatment significantly decreased I/R-induced MDA levels in the uterus by 40–60%, and these levels were comparable with those of the control. Moreover, RSV markedly inhibited I/R-induced MPO activation by 60–80%, and these levels were also comparable with the control (Fig 2B). On the other hand, RSV restored the uterine activity of CAT and SOD close to the levels of the control (Fig 2C and 2D). The results obtained from uterine tissues were consistent with the corresponding results from serum samples in Fig 1.

### 3.2. RSV pre-administration reduces uterine I/R-induced inflammatory responses in the serum and uterus

**Fig 3:**
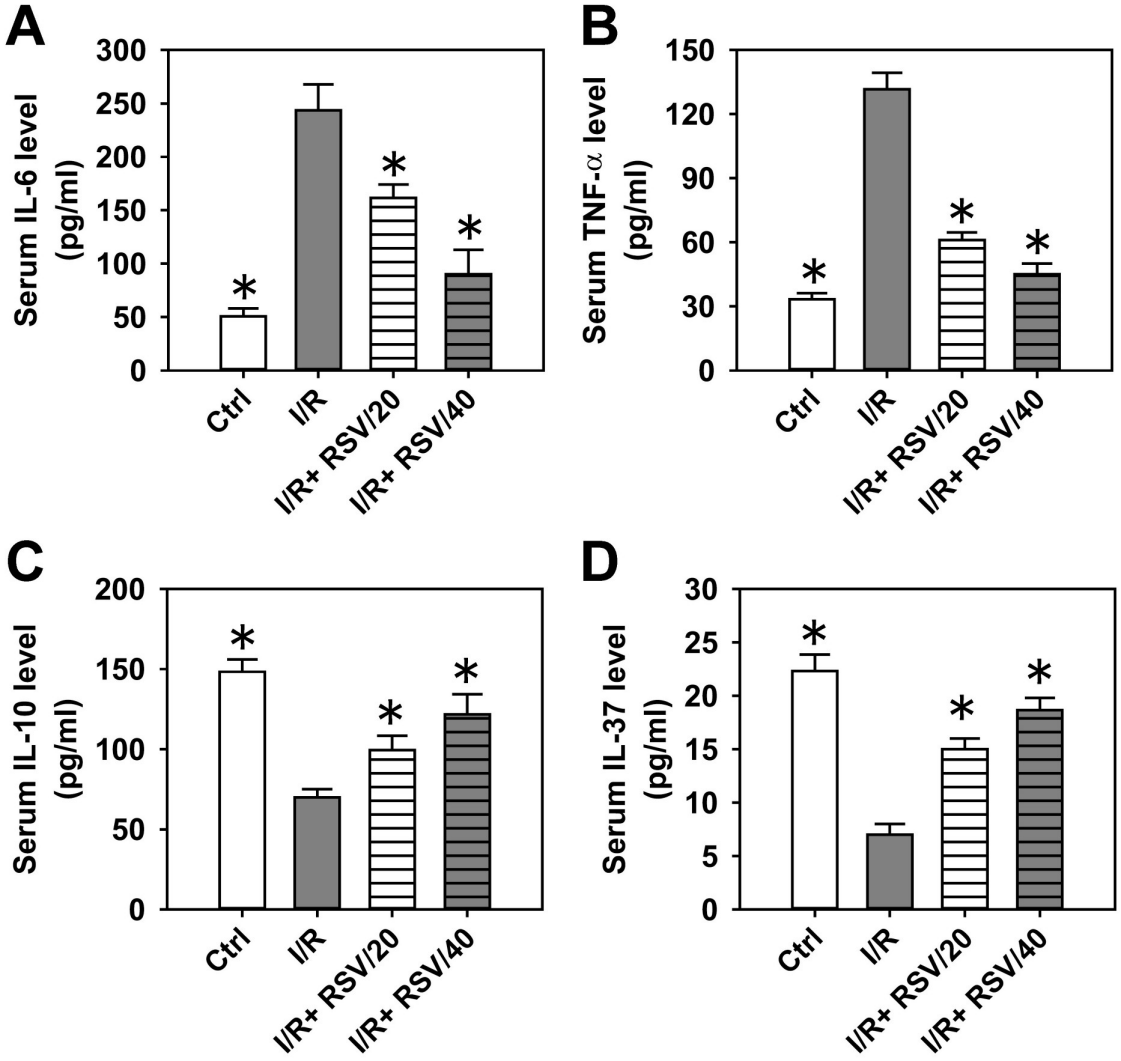
The effect of RSV on the inflammatory response induced by uterine I/R in rat serum. The concentrations of IL-6 (***A***), TNF-α (***B***), IL-10 (***C***) and IL-37 (***D***) in rat serum in control, I/R, I/R+RSV/20, and I/R+RSV/40 groups were examined by ELISA. Bars represent means ± SD, n = 3. *P< 0.05 *vs*. I/R group.

To evaluate the inhibitory effects of RSV on inflammatory responses, the serum levels of pro-inflammatory cytokines IL-6 (Fig 3A) and TNF-α (Fig 3B), as well as the serum levels of anti-inflammatory cytokines IL-10 (Fig 3C) and IL-37 (Fig 3D) were assessed. Results of the ELISA analysis demonstrated that the serum levels of IL-6 and TNF-α decreased by 4-5-fold following I/R injury, while pre-treatment with RSV significantly suppressed IL-6 and TNF-α levels by 60–70% (Fig 3A and 3B). By contrast, I/R decreased both IL-10 and IL-37 serum levels by 50 and 70%, respectively, while RSV pre-administration promoted the upregulation of IL-10 and IL-37 serum levels (Fig 3C and 3D).

**Fig 4:**
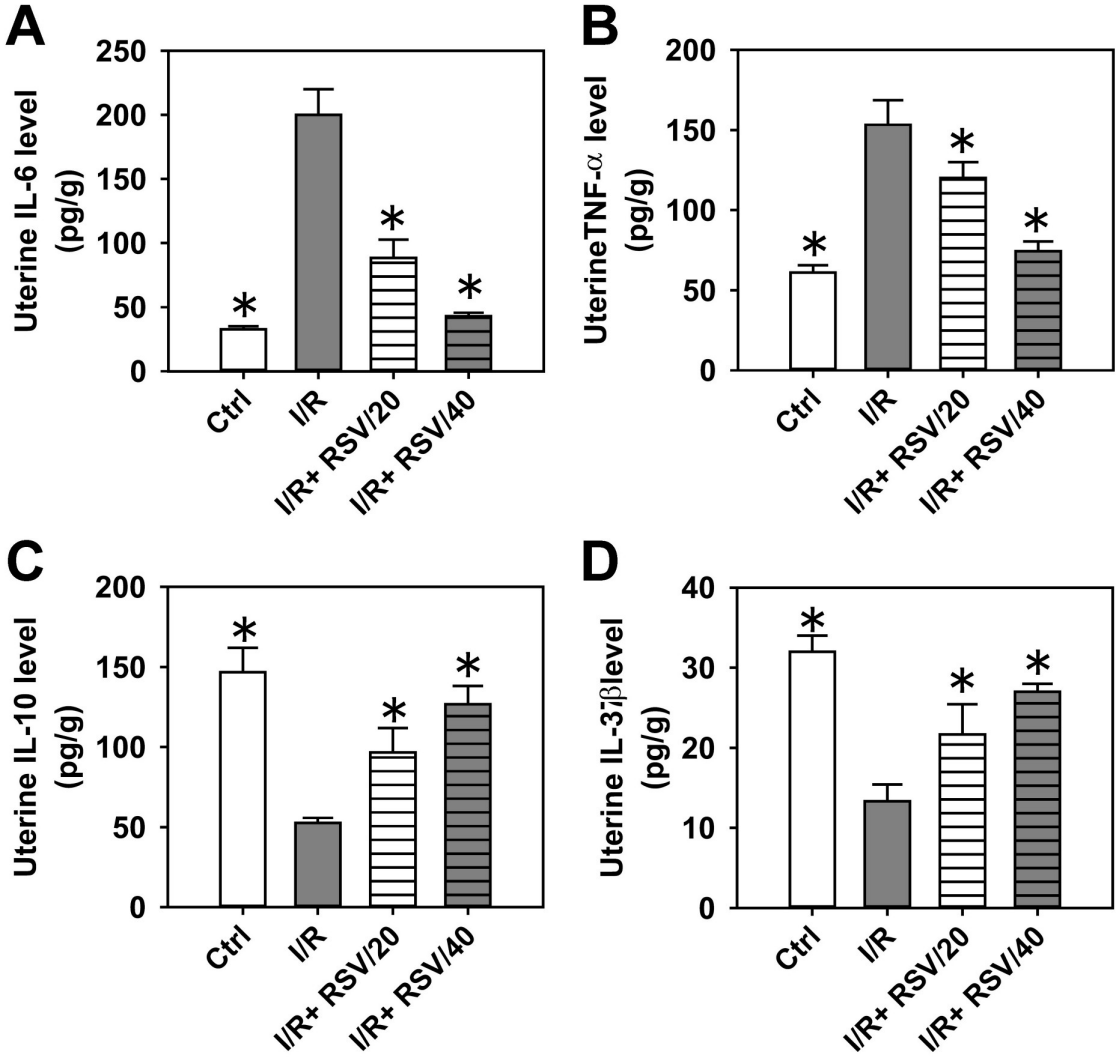
The effect of RSV on the inflammatory response induced by uterine I/R in rat uterus. The concentrations of IL-6 (***A***), TNF-α (***B***), IL-10 (***C***) and IL-37 (***D***) in rat uterus in control, I/R, I/R+RSV/20, and I/R+RSV/40 groups were examined by ELISA. Bars represent means ± SD, n = 3. *P< 0.05 *vs*. I/R group.

Subsequently, the levels of IL-6 (Fig 4A), TNF-α (Fig 4B), IL-10 (Fig 4C) and IL-37 were measured (Fig 4D) in uterine tissues following I/R or I/R+RSV treatment. Results of the present study demonstrated that RSV decreased I/R-induced IL-6 and TNF-α levels by 50–80% (Fig 4A and 4B). On the other hand, RSV restored both IL-10 and IL-37 levels in the uterus, which were inhibited following I/R (Fig 4C and 4D). The results obtained from uterine tissues were consistent with the corresponding results obtained from serum samples in Fig 3.

### 3.3. RSV pre-administration alleviates uterine I/R-induced tissue damage

**Fig 5:**
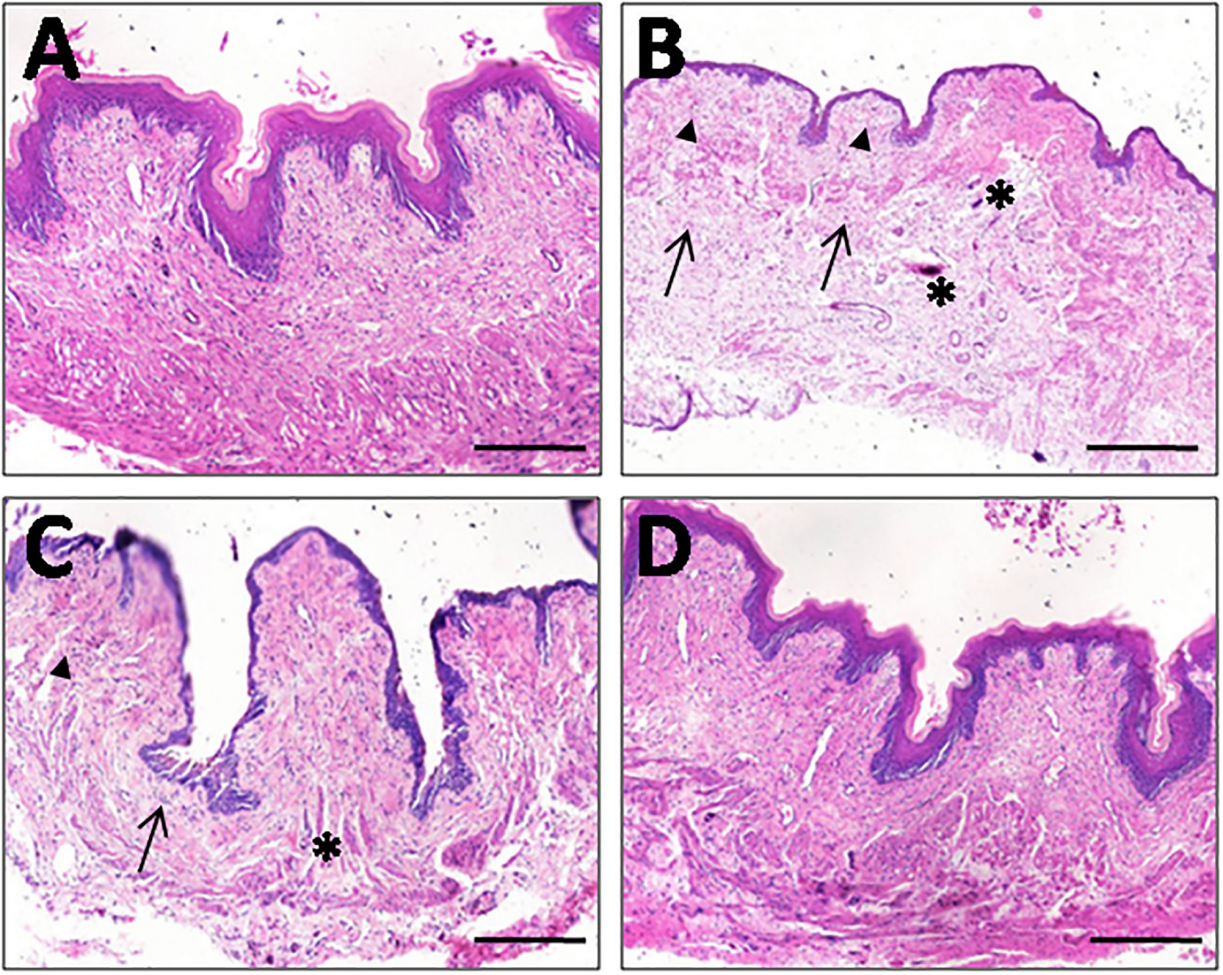
The effect of RSV on I/R-caused uterine damage by H&E staining. (***A***) The control group. (***B***) I/R group. (***C***) I/R+RSV/20 group. (***D***) I/R+RSV/40 group. Arrows: inflammatory cell infiltration. Triangles: stromal cell degeneration. Asterisks: congested vessels. Scar bars = 150 μm. Images are under 100 x magnification.

H&E staining revealed that uterine morphology in the control group displayed a regular structure of high columnar epithelial cells lining the uterine lumen and glands, while the stroma displayed numerous active glands (Fig 5A). In the I/R group, damaged surface epithelial cells, disrupted glandular epithelial cells, increased stromal cell degeneration, as well as vasocongestion in the endometrium and myometrium were observed (Fig 5B). The infiltration of inflammatory cells was also detected (Fig 5B). Compared with the I/R group, RSV groups demonstrated an improved histological appearance, as evidenced by decreased uterine damage and reduced or absent edema (Fig 5C and 5D). Following treatment with a high dose of RSV at 40 mg/kg, the morphology of uterine tissue was comparable with a healthy condition (Fig 5D).

### 3.4. RSV pre-administration activates the PI3K-AKT signaling pathway

**Fig 6:**
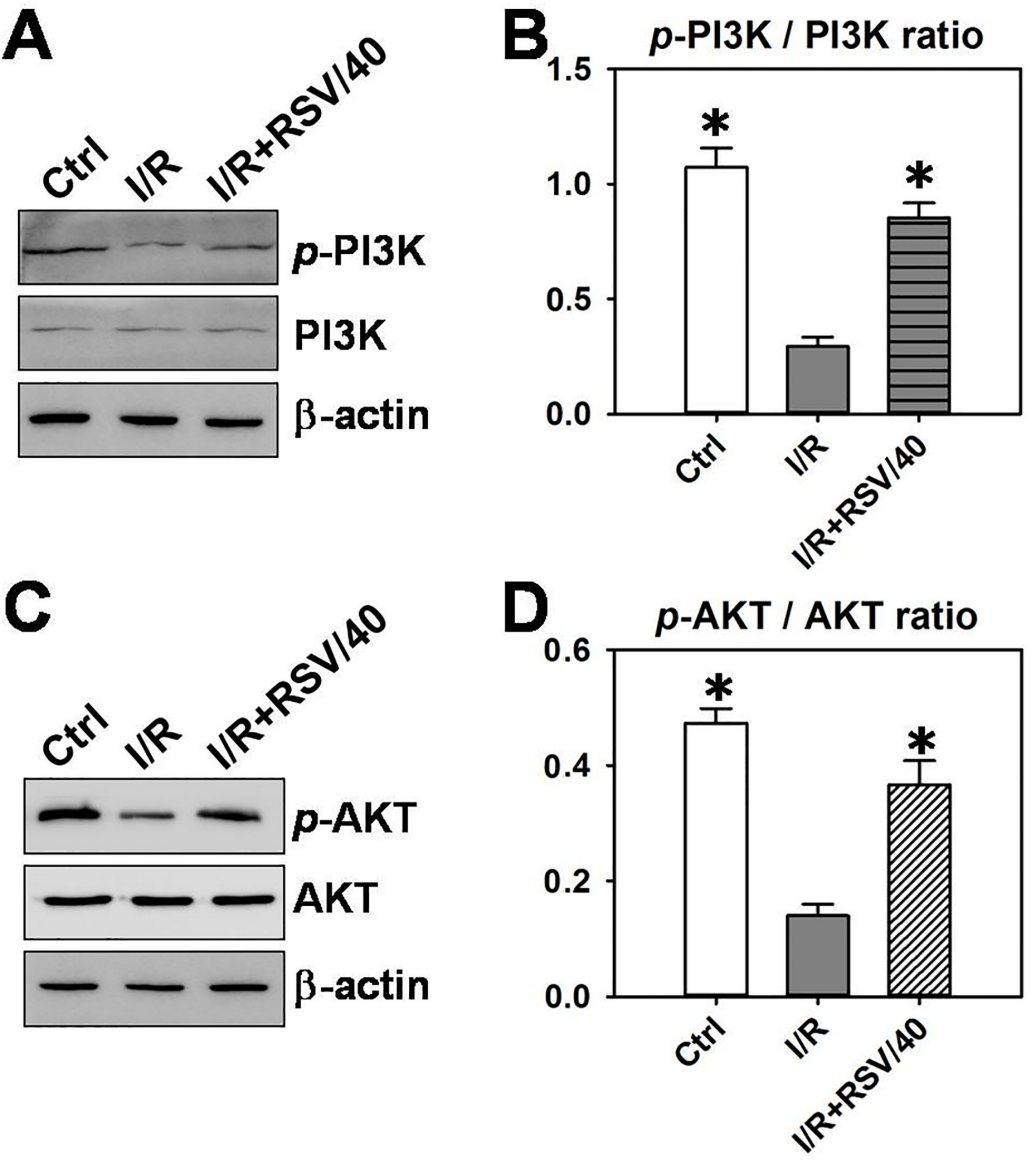
The activation of PI3K-AKT signaling in RSV-mediated protection of uterine I/Rinjury. (***A***) The protein levels of total PI3K and phosphorylated *p*-PI3K in rat uterine tissue in control, I/R, and I/R+RSV/40 groups were assessed by Western blot. (***B***) Densitometric analysis of immunoblotting data in (***A***). (***C***) The protein levels of total AKT and phosphorylated *p*-AKT in rat uterine tissue in control, I/R, and I/R+ RSV/40 groups were assessed by Western blot. (***D***) Densitometric analysis of immunoblotting data in (***C***). Each bar is the mean ± SD, n = 3. *P< 0.05 *vs*. I/R group.

The molecular mechanisms by which RSV inhibits the inflammatory response and oxidative stress during I/R-induced uterine injury were investigated. PI3K-AKT signalling is involved in I/R-induced organ damage [17–19]. However, whether PI3K-AKT is involved in the RSV-mediated protection of uterine I/R injury has not yet been elucidated. Thus, the present study aimed to determine changes in the protein expression levels of PI3K and AKT, as well as their corresponding phosphorylated forms p-PI3K and p-AKT, to determine the activation status of the PI3K-AKT signaling pathway. Results of the western blotting analysis demonstrated that although both I/R and I/R+RSV did not affect the total protein expression of PI3K (Fig 6A) or AKT (Fig 6C), I/R significantly inhibited the expression of p-PI3K (Fig 6A) and p-AKT (Fig 6C) by 70–80%. Moreover, pre-administration of RSV significantly promoted the phosphorylation of PI3K (Fig 6A) and AKT (Fig 6C). Notably, the ratios of p-PI3K/PI3K (Fig 6B) and p-AKT/AKT (Fig 6D) were increased following RSV pre-administration, indicating the activation of PI3K-AKT signaling.

## 4. Discussion

Uterine IR does not only exist during UTx, it also occurs during various gynecological operations and female menstrual cycle. In recent years, the risk of severe bleeding has risen, due to the increasing rate of caesarean-delivered births. The use of tourniquet to strap the lower uterus, creating a temporary ischemia condition, may control the bleeding and help to obtain a clear operation field, which is beneficial for determining the bleeding site [20]. This temporary ischemia also decreases the rate of blood transfusion and the risk of hysterectomy, and is therefore a commonly used strategy to prevent hemorrhage during caesareans [20]. During other complex gynecological operations, such as myomectomy, the temporary blocking of uterine blood flow is also used to reduce bleeding and obtain a clear surgical field [21]. In addition, uterine contraction often occurs under normal physiological conditions, such as menstruation and pregnancy, resulting in the ischemia of uterine cells [22].

Therefore, uterine I/R is an unavoidable problem during and after gynaecological operations. It is also a significant factor causing hysterectomy or infertility due to uterine ischemia-necrosis and poor wound healing [1]. The most fundamental mechanisms underlying I/R injury is the mass production of oxygen free radicals, intracellular calcium overload and the consequent microvascular dysfunction [23]. SOD plays a vital role in the balance of oxidation and anti-oxidation, and in protecting cells from damage. The level of SOD activity directly reflects the ability of the body to scavenge free radicals [24]. MDA is a metabolic product obtained during lipid peroxidation. MDA forms across-linking and polymerized structure with membrane phospholipids and proteins, which leads to changes in the basic characteristics of membranes and the loss of protein activity. The determination of MDA concentration reflects the degree of lipid peroxidation in the body [25], and also indirectly reflects the content of oxygen free radicals and the degree of damage caused by these [26]. In the present study, the activity of SOD was significantly decreased, while the concentration of MDA was significantly increased in the I/R group, indicating that uterine I/R has a direct impact on the metabolic function of uterine cells.

CAT is an antioxidant enzyme predominantly concentrated in the liver. It prevents the formation of the highly reactive hydroxyl radical OH^−^ by converting H_2_O_2_ to H_2_O and O_2_ [27]. Results of previous studies demonstrated that CAT may protect tissues and organs against I/R-induced injury by enhancing their anti-oxidative ability and reducing oxidative stress [28, 29]. In parallel with these results, results of the present study demonstrated that the activity of CAT decreased significantly in the I/R group, while the RSV-pre-treated groups significantly increased the activity of CAT.

RSV, as a natural antioxidant existing in plants, plays an antioxidant role mainly by scavenging the generation of free radicals, inhibiting lipid peroxidation and regulating the activity of antioxidant-related enzymes [30]. Results of a previous study demonstrated that RSV may decrease the levels of peroxylipids, increase the activity of SOD and reduce the water content in rat brain tissues subjected to cerebral I/R, demonstrating a positive protective effect on cerebral I/R injury [31]. RSV administration also reduced the levels of cleaved caspase-3 and MDA, suggesting that RSV may be effective against liver I/R injury by reducing oxidative stress and apoptosis [32]. In addition, RSV demonstrated strong cardio-protective and anti-arrhythmic effects following myocardial I/R, as indicated by the levels of CAT, SOD, MDA and glutathione peroxidase [33]. RSV treatment attenuated lipid oxidation and normalized MDA levels, and improved the histopathology that occurred during renal I/R-induced injury [34]. In parallel with the aforementioned research in different organs and tissues, to the best of our knowledge, the present study is the first to investigate the potential protective role of RSV in uterine I/R injury. The present study was the first to determine that RSV pre-administration significantly inhibited the uterine I/R-induced oxidative stress, as indicated by levels of MDA, CAT, SOD and MPO.

Previous studies have demonstrated that inflammatory responses exert a critical function in I/R-induced organ damage, while inhibition of an inflammatory response may protect organs and tissues against I/R injury [35]. In addition to its anti-oxidative activity, RSV also possesses a potent anti-inflammation property. For example, RSV inhibited TNF-α-induced inflammation to protect against renal I/R injury in diabetic rats [36]. RSV also normalized the deterioration of smooth muscle contractility following intestinal I/R injury in rats by modulating the activity of TNF-α and IL-1α [37]. In addition, RSV was found to delay the loss of retinal ganglion cells and attenuate gliosis-related inflammation in retinal I/R rats [38]. A further study demonstrated that cerebral I/R increased the levels of activated NLRP3 inflammasomes, while treatment with RSV, a specific Sirt1 agonist, attenuated I/R-induced NLRP3 inflammasome-derived inflammation via Sirt1-dependent autophagy induction [39]. Consistent with these results, the present study demonstrated that RSV pre-treatment significantly decreased both the serum and tissue content of pro-inflammatory cytokines, such as IL-6 and TNF-α, while levels of anti-inflammatory cytokines, such as IL-10 and IL-37 were increased. IL-37 is a newly identified member of the IL-1 family, which functions as a fundamental inhibitor of innate immunity and inflammation [40]. IL-37 also plays a protective role against mouse myocardial I/R injury [41]. The aforementioned findings demonstrate that RSV effectively suppresses inflammatory responses subjected to I/R injury.

Results of a previous study demonstrated that the pharmacological effects of RSVare closely associated with the regulation of the PI3K/AKT pathway, which plays a role in tumors, inflammation and injury [17]. Moreover, PI3K/AKT is one of the major signaling pathways involved in MPO-mediated inflammatory responses during ischemic organ injury. A recent study demonstrated that RSV reduced I/R-induced myocardial cell apoptosis and mitochondrial oxidative damage by activating the PI3K/AKT pathway [18]. Another study demonstrated that RSV may also attenuate retinal I/R injury by inhibiting the HIF-1α/VEGF and p38/p53 pathways [19]. In the present study, the expression of p-PI3K and p-AKT in both the serum and uterine tissues in I/R injury rats was significantly reduced, while their expression was restored following intervention with different doses of RSV, indicating that RSV may activate the PI3K/AKT pathway in rats subjected to uterine I/R injury, which could also be the mechanism by which RSV inhibits inflammation and oxidative stress.

In conclusion, to the best of our knowledge, the present study was the first to explore the anti-oxidative and anti-inflammatory effects of RSV in uterine I/R injury. Results of the present study demonstrated that RSV effectively inhibited the inflammatory response, attenuated oxidative stress and activated PI3K-AKT signaling. These results suggest a potential protective effect of RSV against oxidative tissue damage to further improve the antioxidant defense system. However, the present study exhibits a number of limitations. For example, the use of female rats at the same age does not account for differences in the stage of the reproductive cycle, for example proestrus, estrus, metestrus and diestrus, which may impact the histological results. Therefore, further studies are required to confirm the protective effect of RSV during different estrous phrases.

## Supporting information

S1 File (PDF)

S1 Raw images (ZIP)
