# Supplementary material for: Resveratrol inhibits the inflammatory response and oxidative stress induced by uterine ischemia reperfusion injury by activating PI3K-AKT pathway
Source: PLoS One. 2022 Jun 24;17(6):e0266961. doi: 10.1371/journal.pone.0266961 (PMC9231784; doi:10.1371/journal.pone.0266961)
Supplement: S1 File — (PDF) [file pone.0266961.s001.pdf]

EDITORIAL CERTIFICATE

This document certifies that the manuscript listed below was edited for English language, grammar, punctuation, spelling and overall style by one or more editors at Spandidos Publications English Language Editing Service

MANUSCRIPT TITLE:

Resveratrol inhibits the inflammatory response and oxidative stress induced by uterine ischemia reperfusion injury by activating PI3K-AKT pathway

AUTHORS:

Ying Wang et al

SECTIONS EDITED:

Whole manuscript (excluding Title, Author List, Affiliations, Correspondence information, Key words, Declarations, References, Table contents, Table legends, Figure legends)

DATE ISSUED:

08/03/2022

CERTIFICATE VERIFICATION NUMBER:

ESUB-10100

Neither the research content nor the authors' intentions were altered in any way during the editing process. Documents receiving this certification should be English-ready for publication; however, the authors have the ability to accept or reject our suggestions and changes. This certificate may be verified at https://www.spandidos-publications.com/langeediting/publiclanguageediting/certificatedetails?submissionId=10100&uniqueNumber=21d7f36d721d8ec7ba5503eabc3b7be5. If you have any questions or concerns regarding the edited document, please contact Spandidos English Language Editing Service at languageediting@spandidos-publications.com, quoting the certificate verification number. Spandidos English Language Editing provides a high quality range of services for researchers to prepare manuscripts ready for journal submission. For more details regarding our company and current services, please visit: http://www.spandidos-publications.com/.
