# Supplementary figures and images for: Resveratrol inhibits the inflammatory response and oxidative stress induced by uterine ischemia reperfusion injury by activating PI3K-AKT pathway
Source: PLoS One. 2022 Jun 24;17(6):e0266961. doi: 10.1371/journal.pone.0266961 (PMC9231784; doi:10.1371/journal.pone.0266961)

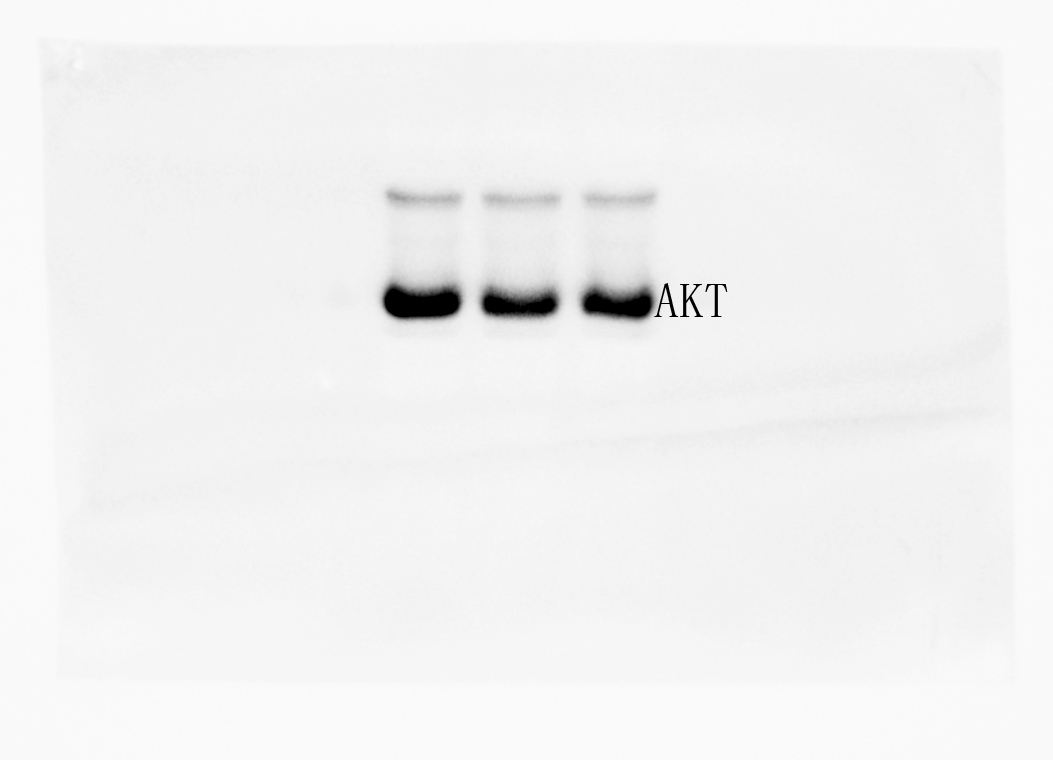

Supplement: S1 Raw images — (ZIP) [file pone.0266961.s002.zip › AKT 6C.jpg]

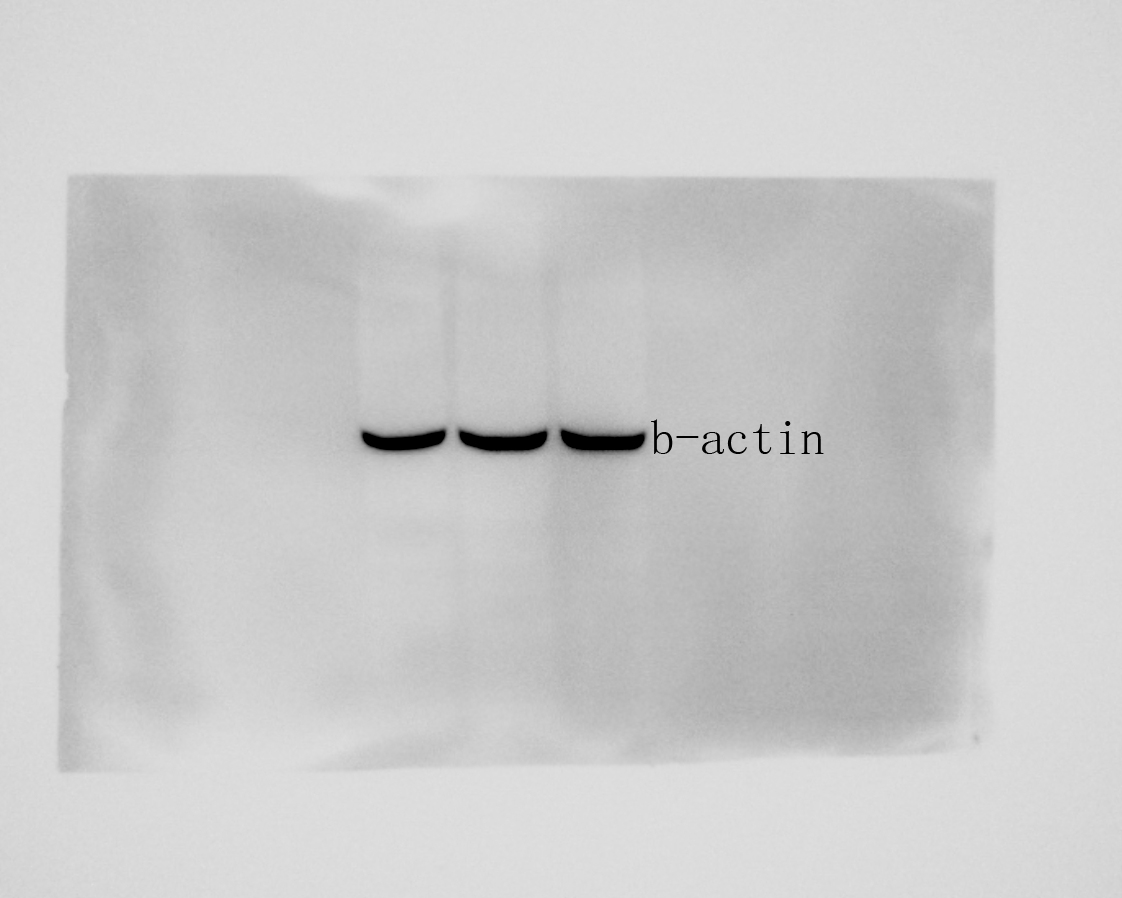

Supplement: S1 Raw images — (ZIP) [file pone.0266961.s002.zip › b-actin 6A.jpg]

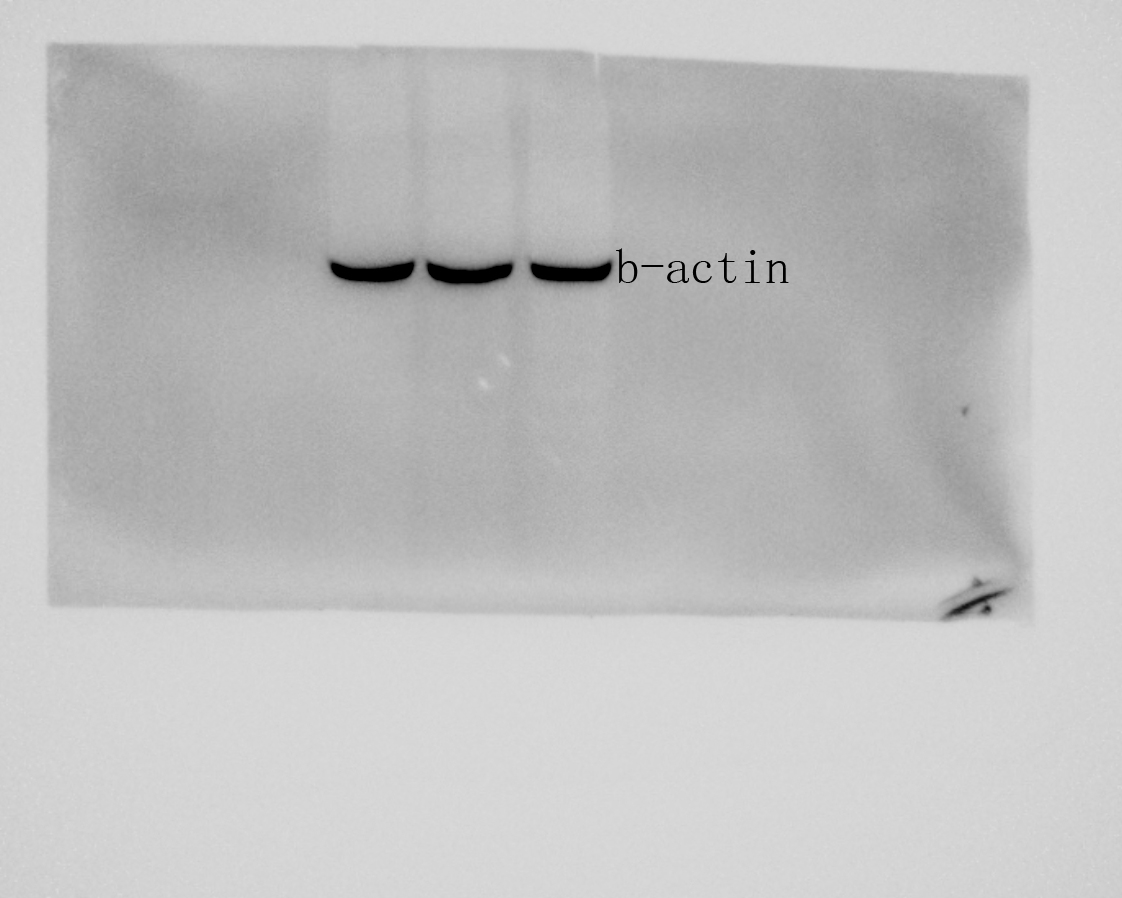

Supplement: S1 Raw images — (ZIP) [file pone.0266961.s002.zip › b-actin 6C.jpg]

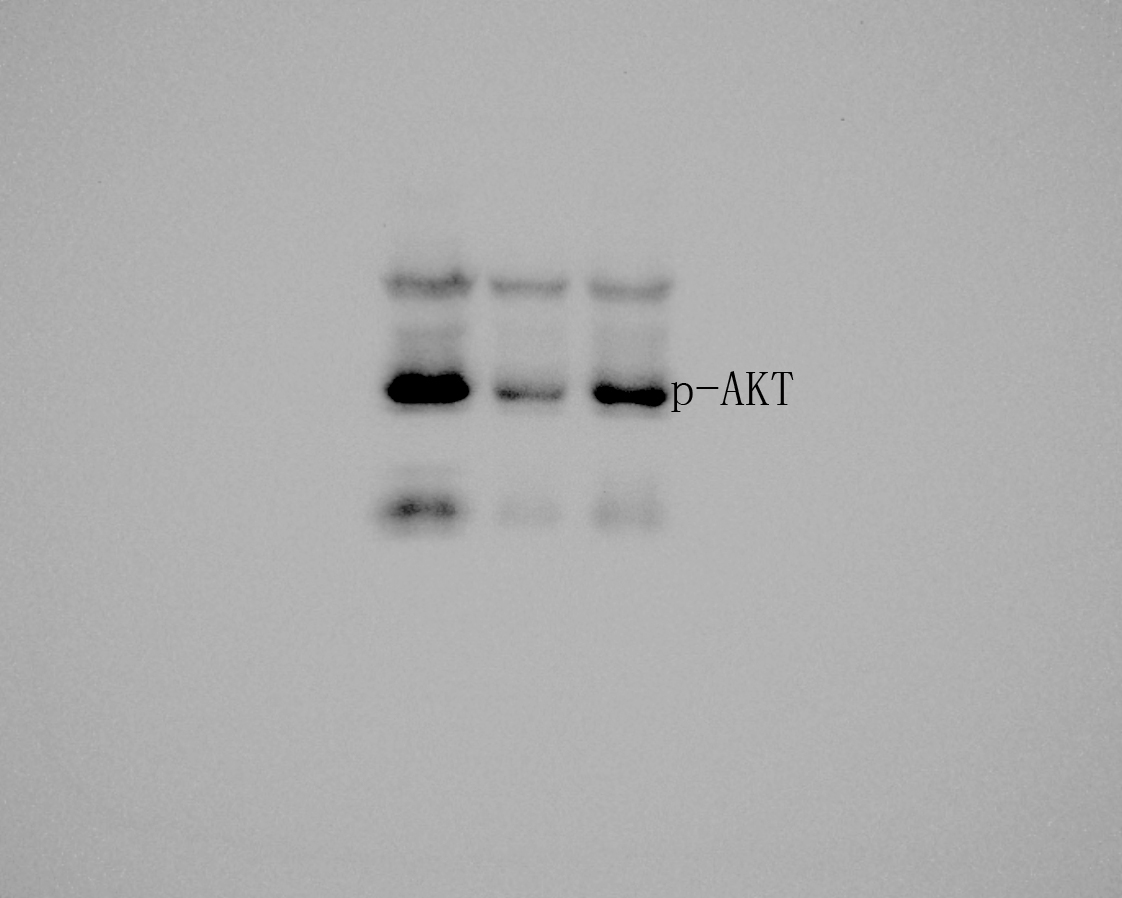

Supplement: S1 Raw images — (ZIP) [file pone.0266961.s002.zip › p-AKT 6C.jpg]

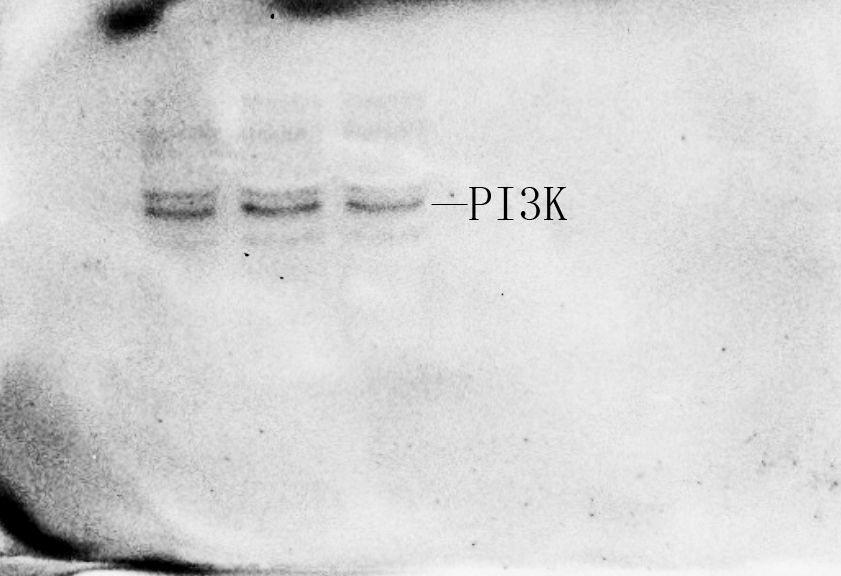

Supplement: S1 Raw images — (ZIP) [file pone.0266961.s002.zip › PI3K 6A.jpg]

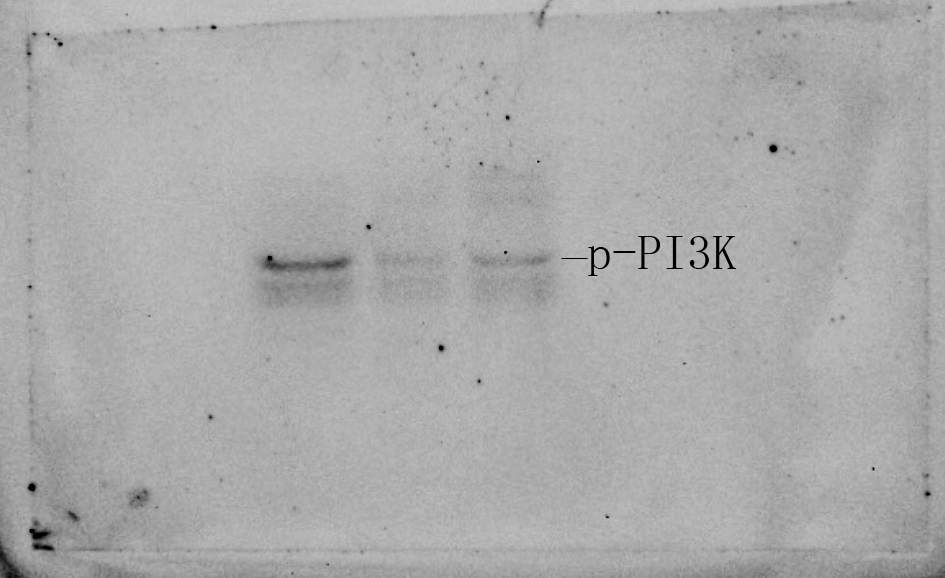

Supplement: S1 Raw images — (ZIP) [file pone.0266961.s002.zip › p-PI3 6A.jpg]

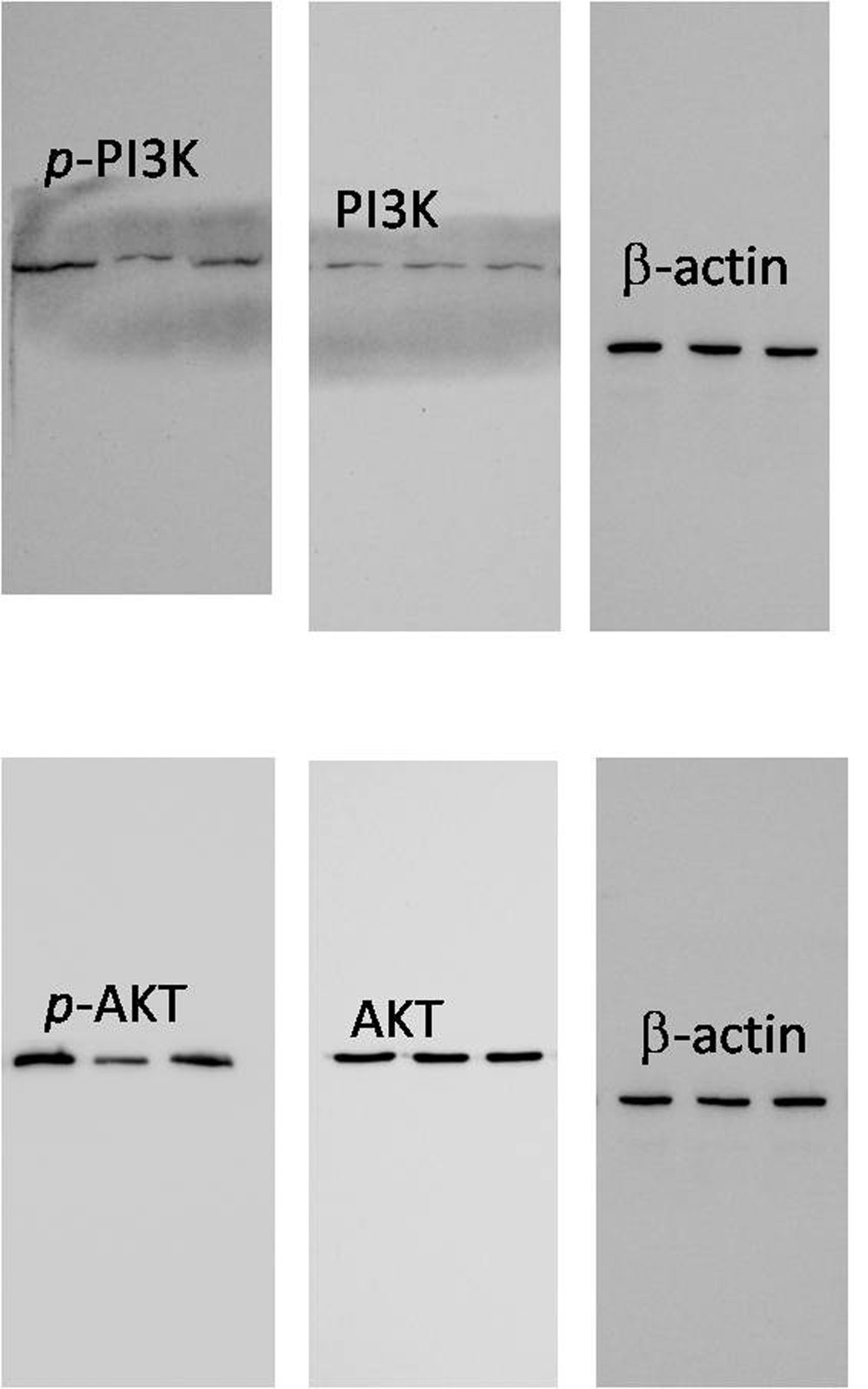

Supplement: S1 Raw images — (ZIP) [file pone.0266961.s002.zip › Raw data.jpg]
